# Corneal cross-linking guards against infectious keratitis: an experimental model

**DOI:** 10.1007/s10792-022-02522-z

**Published:** 2022-10-18

**Authors:** Ayah Marrie, Abdussalam M Abdullatif, Sherief Gamal El Dine, Rania Yehia, Randa Saied, Doaa Ahmed Tolba

**Affiliations:** 1 Ophthalmology Department, Faculty of Medicine, Kasr Al Ainy hospitals, Cairo University, Kasr Alainy Street, Cairo, 11956 Egypt; 2 Microbiology Department, Faculty of Medicine, Kasr Al Ainy Hospital, Cairo University, Cairo, Egypt; 3 Pathology Department, Faculty of Medicine, Kasr Al Ainy Hospital, Cairo University, Cairo, Egypt

**Keywords:** PACK-CXL, Infectious keratitis, Rabbits, Corneal melting

## Abstract

**Background:**

PACK-CXL (photo-activated chromophore for keratitis–corneal cross-linking) is an alternative option in treatment of corneal infections. It inhibits corneal melting by increasing the stromal resistance, besides the microbicidal effect of photo-activated riboflavin.

**Methods:**

Corneal infection with Pseudomonas aeruginosa and Staph aureus bacteria was induced in 20 eyes of 10 rabbits after 6 weeks of corneal cross-linking in half of the eyes, while the other acted as control group. Clinical and corneal histopathological examination was done to evaluate the extent of inflammation, ulceration, organism penetration, and depth of corneal stromal affection.

**Results:**

The control eyes developed severe inflammation compared to the cross-linked eyes. Corneal melting occurred in 6 eyes in the control versus none in cross-linked group. Histopathological examination showed that the inflammation was confined to the superficial part of the stroma with localization of the inflammation in the cross-linked eyes in contrast to the control eyes that showed deep infiltration.

**Conclusion:**

PACK-CXL provides infection localization through increasing the corneal rigidity and resistance to enzymatic digestion, even in the absence of the riboflavin microbicidal role. So, early PACK-CXL is worth to be considered in the IK treatment algorithm.

## Introduction

Infectious keratitis (IK) is the major cause of global corneal blindness [1]. Different microorganisms, including bacteria, fungi, viruses, and parasites, are involved. Broad-spectrum antimicrobial therapy is the usual line of treatment for IK [2]. Unfortunately, the efficacy of antibiotic treatment is declining due to an emerging trend of antimicrobial resistance in ocular infection. Complications, such as corneal melting, perforation, and endophthalmitis, may require additional surgical interventions such as tectonic or therapeutic keratoplasty to save the eye and vision [3–5]. However, performing either tectonic or therapeutic keratoplasty in a “hot eye” is associated with a high risk of recurrence of the disease, secondary glaucoma, and graft rejection or failure [6–9]. These problems drove the need for an alternative to the antimicrobial treatment for IK.

Corneal cross-linking (CXL) using ultraviolet light-A (UV-A) and riboflavin is a technique introduced in the 1990s to stop the progression of corneal ectatic disorders such as keratoconus [10]. It works on increasing corneal biomechanical stability and rigidity. After multiple studies, it showed long-term efficacy and safety for most corneal ectatic disorders [11–13].

Ultraviolet radiation has long been known for its microbicidal effect through the direct damage of the DNA and RNA of different types of microorganisms. In addition, the reactive oxygen species generated from photo-activated riboflavin have a direct effect on the microbial DNA and cell membranes culminating in a strong synergistic antimicrobial action [14–16]. These effects together with the increased corneal rigidity and hence resistance to proteolytic enzymatic digestion of stromal collagen have made CXL an attractive alternative in the management of IK [17]. Initially, it was used to treat advanced infectious melting corneal keratitis. In most cases, stabilization of the melting process and thus avoidance of keratoplasty was achieved [18–20]. Recently, CXL is used to treat early bacterial IK as a first-line treatment without any adjunctive antibiotics with favorable results for most patients [21–25]. At the 9th International Cross-Linking Congress in 2013, with the increase in the number of publications on CXL for the treatment of infectious keratitis, a new terminology regarding this specific use was proposed. It aims to distinguish CXL for infections from CXL for corneal ectasia. The term PACK-CXL: photo-activated chromophore for keratitis–corneal cross-linking was adopted for CXL when treating infectious keratitis [10].

Our aim of the study is to prove the mechanical role of CXL if it guards against the progression of IK and stops organism penetration through increasing corneal rigidity and resistance to proteolytic enzymatic digestion of collagen fibers even in the absence of photo-activated riboflavin microbicidal role.

## Materials and methods

This prospective interventional case–control experimental study was carried out on ten New Zealand albino rabbits of the male sex, obtained from the animal house of Kasr Alainy Hospital, Cairo University. The included rabbits weighed between 1.8 and 2.7 kg and were free from any anterior segment condition (i.e., corneal opacities from previous trauma or keratitis). We excluded any rabbits that had an underlying conjunctival infection (detected clinically or by microbiological analysis of a conjunctival swab). All animals were maintained following the National Research Council's Guide for the Care, ARRIVE guidelines, and the Egyptian guidelines for animal care. The study protocol was approved by the scientific committee of Ophthalmology department at Cairo University. Rabbits were prepped prior to inoculation for one week in “adaptation conditions” (24 °C ± 2 °C, 60 ± 5% relative humidity and 12-h light/dark cycle), one per cage, and fed a laboratory diet. Before bacterial inoculation, a conjunctival swab was obtained to ensure that no bacterial growth was detected and exclude underlying low-grade conjunctivitis was excluded. The animals were anesthetized via intramuscular injections of Ketavet® and Rompun® (each 0.25 mg/kg body weight), in addition to topical benoxinate 0.4%. The right eye of each rabbit had its central 8 mm epithelium removed and cross-linked (CSO Vega CMB X Linker, CSO Scandicci, Firenze, Italy) with riboflavin 0.1% solution drops (10 mg riboflavin5-phosphate in 10 mL of 20% dextran-T-500) applied every 3 min for 30 min, while exposed to UVA (360 nm, 3 mW/cm^2^) for 30 min. This corresponds to a fluence of a total of 5.8 J/cm^2^. After cross-linking, antibiotic OFLOX® (ofloxacin ophthalmic eye drops 0.3%, USA) was applied 3 times daily until complete re-epithelization was achieved. The left eye in each rabbit served as control. The rabbits were kept in the animal house of Cairo University and after a period of 6 weeks, anterior segment optical coherence tomography (ASOCT) (Optovue Inc., Fremont, CA, USA) was done for the cross-linked eye. The next day, bacterial keratitis was induced in the 20 eyes by the inoculation method. Topical corneal anesthesia was achieved by instilling a drop of benoxinate hydrochloride 0.4%. Isolates of Staphylococcus aureus and Pseudomonas aeruginosa were obtained from the medical microbiology and Immunology department at Cairo University. The MRSA strain ATCC 33592 was isolated from blood samples, and the MDR Pseudomonas aeruginosa ATCC (BAA-2108) strain was isolated from sputum samples. They were both obtained from the ATCC (American Type Culture Collection (ATCC, Manassas, VA, USA). We used both organisms as they have been shown to be the most common organisms in several IK studies [26]. The isolates were identified by standard bacteriological techniques. Suspensions of various isolates were prepared by culturing a specimen (i.e., a single colony) of each isolate, which was picked up from a pure culture on a plate with a standard loop and placed into 10 mL of nutrient broth. These isolates were then incubated for 24 h at 37 °C. Specimens from the bacterial suspensions were taken with the standard loop and inoculated onto each animal’s abraded cornea after creating linear epithelial injuries with a sterile 21-gauge needle was used to. Intrastromal injection of a suspension of pure strain of P. aeruginosa was applied directly in the stroma of 10 eyes of 5 rabbits, while the other 10 eyes were injected with S. aureus bacteria. Slit lamp examination was done prior to infection and every 12 h post-infection until killing. The examination was scored according to the grading system described by Hobden et al. [27]. Briefly, scores of 0 (absent) to 4 (severe) were assigned to seven parameters: conjunctival injection, conjunctival chemosis, iritis (cell and flare), fibrin in the anterior chamber, hypopyon, stromal infiltrate, and stromal edema. The sum of the scores from each of the seven parameters reflects the degree of inflammation observed. So, the highest possible score is 28. The rabbits were killed within 72 h with the progression of the severity of infection in the control eyes.

The specimen was preserved in 10% buffered formalin and then stained with hematoxylin and eosin and examined for evidence of inflammation in the bulbar conjunctiva, the meibomian glands, and the cornea. Histological examination was done for the extent of inflammation, epithelial changes, ulceration, organism penetration and the thickness of corneal stromal affection.

## Results

### Prior to infection

**Fig. 1:**
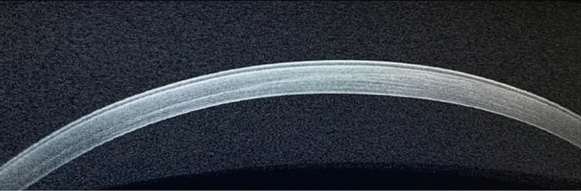
ASOCT of a cross-linked rabbit cornea with evident demarcation line

Following CXL, all epithelial defects healed within 2–5 days. ASOCT at week 6 showed an evident demarcation line (DL) at the anterior third to two thirds of the cornea as the example shown in Fig. 1. Before induction of infection, all rabbits had normal corneas with zero scores of inflammations.

### Post-infection

#### Clinical examination

**Fig. 2:**
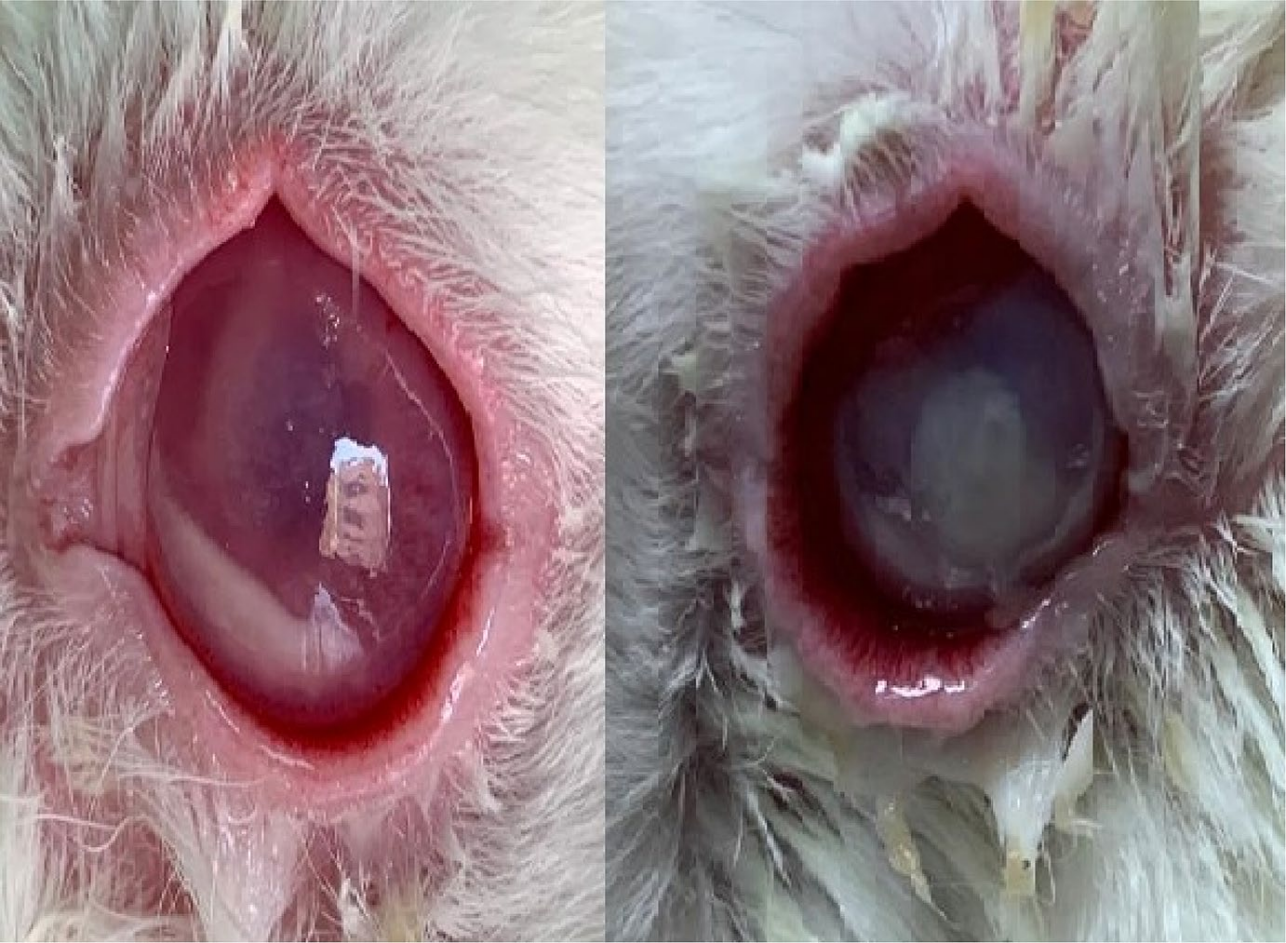
Images showing the clinical manifestations of less severity in the cross-linked eye (left) versus control eye (right) after 72 h of inception of infection

After 24 h of infection, all rabbits started to develop mild conjunctival injection and keratitis with superficial ulceration. Then, the inflammation in all eyes starts to worsen at a different pace. Notably, the control group without cross-linking developed the severest inflammation that necessitated killing after 72 h as shown in Fig. 2. Corneal melting occurred in 6 eyes in the control group versus none in cross-linked group. Table 1 shows the progression of Hobden score in the three groups over the 72 h.

**Table 1:** Hobden clinical score of the 3 groups

Group	Post-infection slit lamp examination score				
	24 h	36 h	48 h	60 h	72 h
Pseudomonas aeruginosa (group 1A)	3.2 ± 0.45	6 ± 0.70	6.8 ± 0.83	8 ± 1.00	9 ± 1.41
Staph aureus (group 1B)	3 ± 0.70	5.8 ± 0.83	6.4 ± 0.54	7.4 ± 0.54	8.8 ± 0.83
Control (group 2)	7.6 ± 1.14	14 ± 3.39	18 ± 2.23	22.4 ± 2.30	27.6 ± 0.54

#### Pathological examination

Pathological examination results showed that the ulceration, inflammation, and stromal affection were less in cross-linked subgroups (groups 1A, 1B) compared to the control (group 2).

**Fig. 3:**
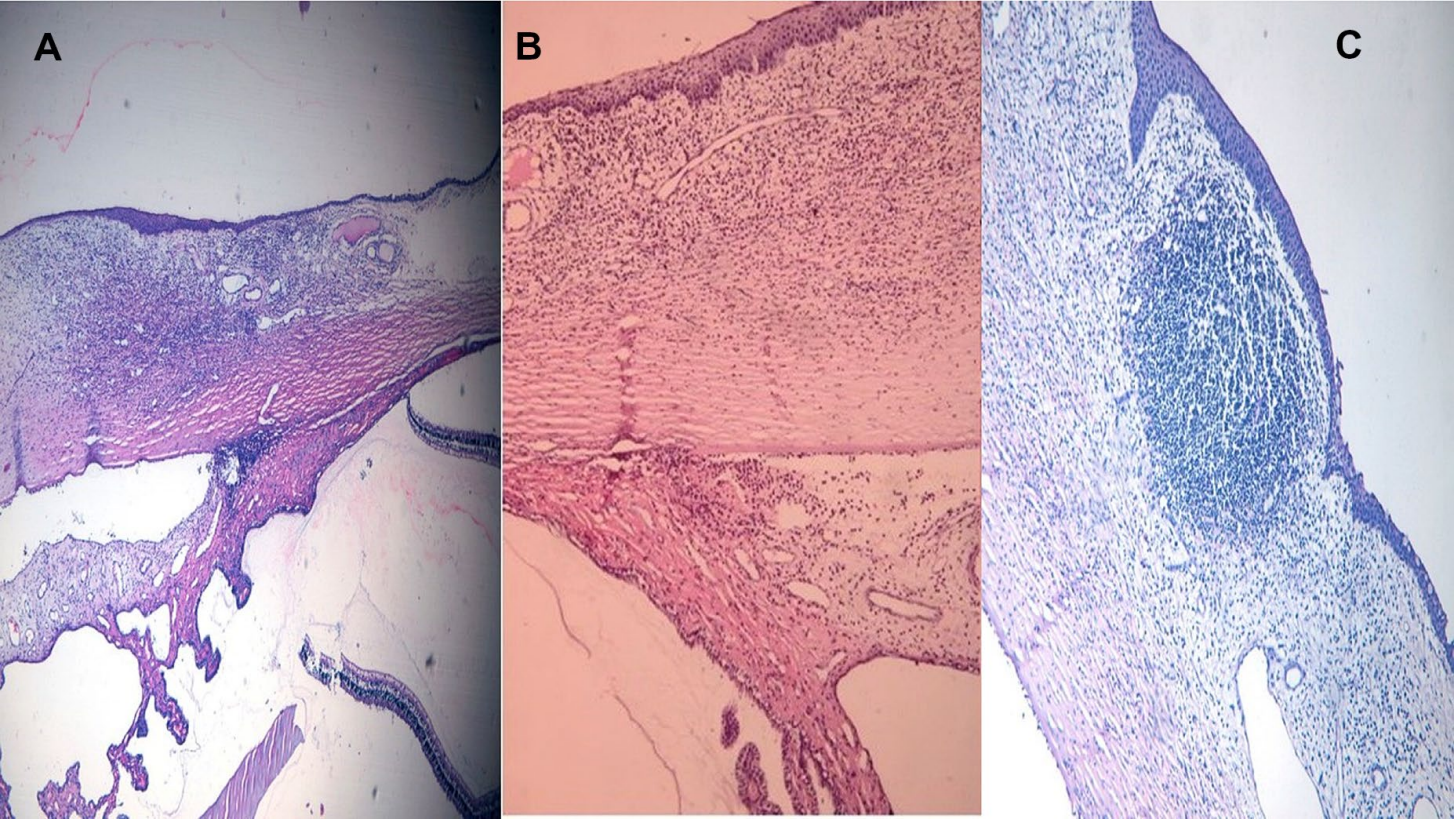
(H&E, X400 objective lens): **A** Minimal inflammatory cellular infiltrate predominantly lymphocytes with intact epithelium and minimal affection of iris and ciliary processes in an example of the cross-linked group. **B** Infiltrate of inflammatory cells with lymphocytic aggregation limited to the superficial stroma. **C** infiltrate predominantly lymphocytes underneath intact corneal epithelium with localization of inflammation in three different examples of the cross-linked group

Cross-linked eyes (groups 1A, 1B) showed that the infiltration of the inflammatory cells and the lymphocytic aggregation were confined underneath the corneal epithelium in the superficial part of the stroma with localization of inflammation and intact epithelium in the upper 30–50% of the stroma. There was minimal affection of iris and ciliary processes as shown in Fig. 3.

**Fig. 4:**
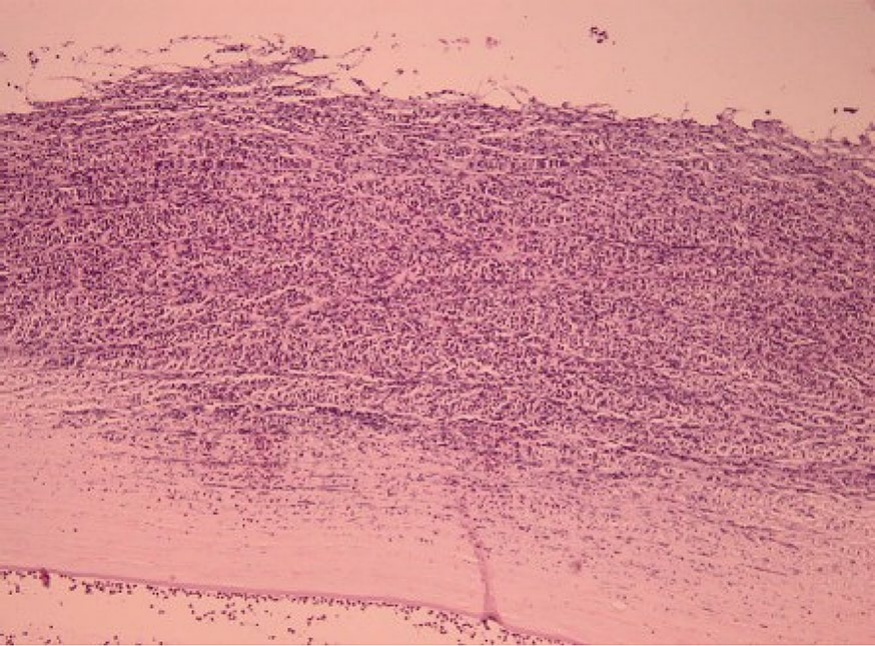
(H&E × 200 & 400 objective lens) Marked corneal abrasion with dense inflammatory infiltrate with aggregation in the anterior chamber in an example of the non-cross-linked group

The control groups (group 2A, 2B) without cross-linking showed marked corneal abrasions and erosions with dense inflammatory infiltrates digesting the basement membrane, invading most of the stromal depth more than 75% of the specimens and with lymphocytic aggregation reaching the anterior chamber. There was more evident affection of iris and ciliary processes as shown in Fig. 4.

## Discussion

Recently, evidence is accumulating proving that PACK-CXL has become a possible effective alternative to standard current antibiotic therapy in treating corneal infections. It may reduce the worldwide burden of microbial resistance to therapeutic agents [28]. That is why for the past years, researchers have started paying more attention to studying its efficacy in IK treatment with variable severity. In this study, we tried to prove the long-term mechanical effect of CXL in localizing the infection to the anterior stroma hindering the penetration of the organisms. This was applied by induction of infection 6 weeks after CXL procedure in an experimental rabbit model. Thus, we assume that the antimicrobial effect of riboflavin is abolished.

The clinical manifestations of both P. aeruginosa and S. aureus IK were markedly decreased by the prior CXL procedure. Additionally, the microscopic findings came to confirm the limitation of the infection and its consequent inflammation to the anterior portion of the stroma confirming the mechanical role of CXL in halting infection. This acts by creating additional chemical bonds inside the corneal stroma and photo-polymerization that increases the rigidity of the corneal collagen [29].

Many studies proved the efficacy of PACK-CXL in S. aureus IK as Tal et al. reported that CXL had a curative effect both as a stand‐alone treatment and in combination with medical therapy [30]. Also, Kozobolis et al. reported favorable clinical outcomes after PACK-CXL in 2 patients with combined bullous keratopathy and ulcerative keratitis [31].

The progression of corneal melting is influenced by the elaboration of excessive tissue degradative proteases [32, 33]. One group of these proteases is the matrix metalloproteinases (MMPs). MMPs are regularly shown to have a role both in infectious and non-infectious causes of corneal tissue destruction. In this study, we reported a reduction in corneal melting complications. Similarly, early studies by Iseli et al. reported regression of the corneal melting process after PACK-CXL in 5 patients with therapy-resistant bacterial or fungal ulcerative keratitis [34]. Also, Makdoumi et al. used PACK-CXL as primary therapy in patients with bacterial keratitis and reported prevention of progression of melting in all cases [35]. In addition, Panda et al. used PACK-CXL to treat patients with antimicrobial-refractory associated corneal melting. The melting regressed, and emergency keratoplasty was avoided in all 7 eyes [36]. PACK-CXL was proven also to be an alternative to antimicrobial drugs for first-line or only treatment of early to moderate infectious keratitis of bacterial, fungal or mixed microbial origin by Hafezi et al. through a multicentered trial published early in 2022 [37].

Treatment with PACK-CXL halts corneal melting and improves infectious keratitis through at least 2 mechanisms, and these probably work in synergy. First, it is well established that pathogens that cause corneal melting may act by enzymatic digestion [38, 39]. Because PACK-CXL increases tissue resistance to enzymatic digestion, the cross-linking procedure may help the corneal stroma resist proteolysis by enzymes from polymorphonuclear leukocytes participating in the inflammatory process [40]. A fortified stroma also may block the penetration or the effect of toxins from the pathogenic organism. Second, the phenomenon of apoptosis induced by PACK-CXL likely not only kills keratocytes but also kills microbes, which decelerates the infectious process [41]. Previous studies supported this latter antimicrobial mechanism. However, in this study, we can support the first assumption as we abolished the antimicrobial effect of riboflavin with very favorable results.

On the other hand, the positive outcomes obtained in this study are opposed by others as Prajna NV et al. reported that patients who underwent CXL were no more likely to re-epithelialize by 3 weeks or 3 months, they had no improvement in infiltrate or scar size, and they even had worse final visual acuity [42]. Also, some published studies reported that the PACK-CXL procedure is not effective in managing fungal keratitis [43, 44]. Their negative results could be attributed to the fact that a sufficient concentration of riboflavin in corneal stroma is crucial to get a biomechanical effect of corneal CXL [45, 46]. Furthermore, the majority of earlier research focused on resistant kinds of ocular infections in which antimicrobial drugs were given for long periods and in which the infections had failed to resolve. In these instances, the PACK-CXL strategy was not applied until the infections had advanced to a late stage and significantly damaged the cornea [47]. As these studies did not elucidate the baseline ulcer size and the ultraviolet (UV) fluence setting employed in their study, Hafezi et al. again refuted their theory. Given that the Dresden protocol was their presumptive method, it may not be sufficient for treating infectious keratitis, whether it is bacterial or fungal in origin, as it was developed for ectatic corneas guaranteeing safety in order to protect corneal endothelium cells [48].

Similar to previous reports on the efficacy of PACK-CXL in IK treatment, we conclude that PACK-CXL provides satisfactory limitation of infection and resistance to the proteolytic effect against melting. The inclusion of early PACK-CXL treatment in the IK treatment algorithm is considered to be appropriate in localizing the tissues’ inflammation and damage. However, we did not do any microbiological evaluation of the bacterial burden as colony-forming unit (CFU) in the follow-up which is a limitation in this study.
